# Social prescribing for older people and the role of the cultural sector during the COVID‐19 pandemic: What are link workers' views and experiences?

**DOI:** 10.1111/hsc.13949

**Published:** 2022-07-23

**Authors:** Stephanie Tierney, Caroline Potter, Kathryn Eccles, Oluwafunmi Akinyemi, Jordan Gorenberg, Sebastien Libert, Geoff Wong, Amadea Turk, Kerryn Husk, Helen J. Chatterjee, Emma Webster, Beth McDougall, Harriet Warburton, Lucy Shaw, Kamal R. Mahtani

**Affiliations:** ^1^ Nuffield Department of Primary Care Health Sciences University of Oxford Oxford UK; ^2^ Nuffield Department of Population Health University of Oxford Oxford UK; ^3^ Oxford Internet Institute University of Oxford Oxford UK; ^4^ Peninsula Medical School University of Plymouth Plymouth UK; ^5^ Division of Biosciences University College London London UK; ^6^ Gardens, Libraries and Museums University of Oxford Oxford UK

**Keywords:** COVID‐19, cultural sector, link workers, older people, questionnaire, social prescribing

## Abstract

Older people's well‐being can be bolstered by engaging with cultural activities and venues. They may be encouraged to try cultural offers by a link worker as part of social prescribing. However, the cultural sector, like all parts of life, was affected by the COVID‐19 pandemic; this has had implications for cultural offers available to link workers. A study was conducted to explore the views and experiences of link workers in using the cultural sector within social prescribing, particularly for older people (aged 60+) during the pandemic. An online questionnaire was distributed to and completed by link workers in the UK. Data were analysed mainly using descriptive statistics. Open text responses were clustered into similar ideas to create key concepts. Useable responses were received from 148 link workers. They highlighted a general lack of interaction between link workers and the cultural sector about how the latter could support social prescribing. Results suggested that personal familiarity with cultural offers might prompt link workers to refer to them. Some respondents proposed that cultural offers were regarded as elitist, which deterred them from referring there. However, there was a general acknowledgement that the cultural sector could contribute to social prescribing. Link workers need to regard the cultural sector as accessible, appropriate, adequate, affordable and available before referring older people to cultural offers as part of social prescribing. Link workers may benefit from becoming more familiar with cultural sector staff and offers, including online resources, so they can then propose them to patients with confidence.

**What is known about this topic?**

- The cultural sector can support health and well‐being.
- It has been used as part of social prescribing, to assist with things like loneliness.
- Cultural offers specifically aimed at older people have been designed.

**What this paper adds?**

- Interaction between link workers and the cultural sector about how the latter could support social prescribing is lacking.
- Familiarity with cultural venues appears to prompt link workers to refer to them.
- Cultural offers/venues are regarded as elitist by some link workers, which can prevent them from referring older people.

## 1 INTRODUCTION

Social prescribing recognises that health outcomes may be directly or indirectly linked to non‐medical difficulties such as deprivation, social isolation, housing or unemployment (Buck & Ewbank, 2020). It involves connecting people to community groups or organisations to help with these difficulties. It encourages a move away from silo working (i.e. fragmented support from a range of health and care services that operate independently of each other), towards appreciating and utilising the strengths of multiple, diverse sectors. It offers the promise of interdisciplinary collaboration across health, social care and the voluntary‐community sector, and involves developing personalised solutions to individual difficulties (NHS England, 2020).

Link workers (also known by other titles such as community connectors or navigators—Tierney et al., 2019) are a key part of the social prescribing pathway (Frostick & Bertotti, 2021). They help people to identify factors impeding their health and well‐being, and co‐develop plans to access groups, organisations, charities or activities in the local area to address these issues (NHS England, 2019). Link workers can help to activate an individual's self‐reliance or agency; this may stop someone turning immediately to their GP for assistance (Tierney et al., 2020; Tierney et al., 2022). Link workers can also help people to feel better connected, giving wider purpose to their life, although positive outcomes are not inevitable (Husk et al., 2020).

Social prescribing schemes vary in how they are delivered, duration of support and their target populations (Younan et al., 2020). They can be provided to children, adults and older people (Cartwright et al., 2022). For older people, in particular, social prescribing offers a useful means of addressing social, emotional or practical needs that can arise due to ageing, as individuals become more susceptible to long‐conditions and to being isolated (Hamilton‐West et al., 2020). Older people may also encounter sensory impairment (e.g. visual and/or hearing) that affects their social connections, mobility and overall well‐being (Vogelpoel & Jarrold, 2014). It has been suggested that through social prescribing, older people can experience improvements such as increased self‐esteem and decreased loneliness (Bertotti et al., 2018).

Several researchers have explored the impact of COVID‐19 on social prescribing (e.g. Fixsen et al., 2022; Morris et al., 2022; Westlake et al., 2022), a time when link workers had to be agile and open to change. During the first half of 2020, conversations that link workers had with patients often focused on immediate needs—such as food and medical supplies, or information on guidelines to stop the spread of COVID‐19. These conversations had to be carried out remotely rather than face‐to‐face. It could be harder to develop a rapport online or by telephone, although there were reports of some individuals engaging and being more open with link workers when communicating remotely (Morris et al., 2022).

At the time of the first lockdown, many link workers in primary care had just started in their role so lacked the opportunity to develop strong community links. The situation was compounded by existing services and activities temporarily closing due to social distancing measures, placing a limit on what link workers could connect people to for support (Westlake et al., 2022). This was a problem since success for link workers relies on connecting people to community assets that meet their non‐medical difficulties (Tierney et al., 2020; Tierney et al., 2022); to do this, link workers ideally need to know about a range of local groups, activities, organisations and charities.

The cultural sector forms part of link workers' arsenal of community assets that they can refer people on to. A subset of or companion to social prescribing includes things like arts‐on‐prescription (Bungay & Clift, 2010; Poulos et al., 2019) and museums‐on‐prescription (Deakin, 2022; Thomson et al., 2018). These schemes vary but what unites them is the use of art, creative or other activities to improve health and well‐being. It usually involves referral to a cultural or heritage organisation—connecting people to support outside of traditional medicine that complements more conventional approaches to health (Culture Health and Wellbeing Alliance, 2021). Health and well‐being benefits experienced from engaging with the cultural sector have been reported (All‐Party Parliamentary Group on Arts, Health and Wellbeing, 2017; Fancourt & Finn, 2019). However, link workers' views and experiences of the cultural sector within social prescribing have been under‐researched.

The authors were funded by the Arts and Humanities Research Council (AHRC) to study the following question: *Cultural institutions as social prescribing venues to improve older people's well‐being in the context of the COVID‐19 pandemic: What works, for whom, in what circumstances and why?* To make the research manageable within a 12‐month period, the study focused on specific cultural venues or settings—museums, libraries and public or curated gardens. The overall study had three components—a review, interviews and a questionnaire. This paper reports on the last of these. The questionnaire was completed by link workers online. It asked about their knowledge and experiences of the cultural sector. A copy of the questionnaire and details of the broader research can be found on the study's webpage (https://socialprescribing.phc.ox.ac.uk/research/projects/social‐prescribing‐for‐older‐people‐in‐the‐time‐of‐covid‐drawing‐on‐the‐cultural‐sector).

## 2 METHOD

### 2.1 Design

A cross‐sectional survey to explore the views and experiences of link workers in using the cultural sector within social prescribing, particularly with older people (aged 60 and above). The Oxford University Central Research Ethics Committee approved the study (R73809/RE002).

### 2.2 Participants and procedure

Link workers based in the United Kingdom (UK) were asked to complete an online questionnaire during April–May 2021. They were recruited mainly through the Social Prescribing Network (www.socialprescribingnetwork.com/) and the National Association of Link Workers (www.nalw.org.uk/). These organisations sent out a short advertisement (written by the researchers) to those on their mailing lists. It contained a link to the questionnaire. Snowball sampling was also employed; members of the research team asked link workers they knew personally to send the link on to colleagues. A mixture of closed and open‐ended questions was developed following (a) a literature review that was conducted on the topic by the project team (Tierney et al., 2021) and (b) stakeholder consultations (Webster, 2021). The online questionnaire was piloted with three link workers; changes were made based on their feedback. Questions centred on the following:

- Personal use by link workers of the cultural sector for health and well‐being;
- The meaning of ‘culture’ to link workers;
- Link workers' use of the cultural sector within social prescriptions, especially for older people;
- Link workers' knowledge of cultural sector provision that they could draw upon for social prescribing;
- Link workers' views of the cultural sector as part of a social prescription, especially for older people.

The questionnaire's introduction explained the project. It asked participants to confirm they were a link worker based in the UK and consented to participating in the study by ticking a box.

### 2.3 Data management and analysis

Online survey software provided by JISC (www.jisc.ac.uk/online‐surveys) was used to create the questionnaire and to collate responses. Data were exported into Excel and were then formatted for importation into SPSS (version 27), a statistical software package. Analyses were primarily descriptive (i.e. frequencies of responses). In addition, exploratory analyses were conducted using inferential statistics based on queries from stakeholders who were consulted whilst undertaking the project. Chi‐square tests for independence were used to explore hypotheses about how likelihood of referral to a cultural sector venue might vary according to the following link worker characteristics:

- *Length of time in post as a link worker* (hypothesis: those longer in post would have greater knowledge about the availability of resources and, therefore, might be more likely to refer to a garden, library or museum);
- *Personal experience of using gardens, libraries or museums* (hypothesis: link workers with personal experience of these venues would be more likely to refer people to them);
- *Interaction between link workers and staff from gardens, libraries or museums* (hypothesis: link workers who reported approaching these venues about social prescribing offers, or who were approached by these venues about such offers, would be more likely to refer to them).

Free‐text responses were prompted by open‐ended questions. With these data, two researchers (S.T. and A.O.) undertook an inductive analysis, looking for patterns and unique suggestions. First, they read a set of comments, identifying key concepts across what respondents had written for a particular question. They then colour‐coded responses based on these concepts. After colour coding a set of comments, to highlight ones that were on a similar issue, they were able to write a narrative that incorporated the range of responses expressed to a particular question.

## 3 RESULTS

Responses were provided by 148 link workers; all were included in the analysis. Two thirds were between 41 and 60 years of age, and most were female (93%) and White British (89%). Time as a link worker ranged from less than 6 months (15%) to more than 4 years (11%). They came from across England. Scotland was also represented (1% of respondents), but no‐one from Wales or Northern Ireland completed the questionnaire. Of respondents, 45% were employed by a Primary Care Network (PCN), 32% by the voluntary‐community sector, and the remainder said they worked for another organisation (including social enterprises and charities).

Data from the questionnaire, especially open comments, highlighted issues related to accessibility and use of the cultural sector by link workers. Accessibility is a term that can be viewed from a number of perspectives. A classification that derives from the European Patients Forum (2016) was used to structure the presentation of results; it was selected because it is relatively comprehensive and is based on the priorities of those receiving healthcare. It has the following five core components:

- Adequate: *What is the quality of the cultural offer? Does it meet older people's expectations? Is it serving a purpose?*
- Accessible: *Is the offer described in a way that is clear to older people? Is it delivered in a way or setting that people can access? Do link workers know what is on offer?*
- Affordable: *Are there costs (financial, social) associated with an offer?*
- Available: *Is the offer provided in a timely manner? Is access equitable?*
- Appropriate: *Does the offer meet an older person's needs in a way that is respectful and sensitive?*

The remainder of the results section uses broad headings to present key concepts from the data, particularly in relation to accessibility (as delineated above).

### 3.1 Personal meaning and use of cultural venues or activities

Respondents' descriptions of culture or the cultural sector varied (see Table 1). Some perceived such venues as *“elitist”, “high brow”, “costly”*. Others alluded to culture more positively in terms of community and belonging. Meaning could be shaped by personal experience of cultural venues and activities. In terms of gardens, libraries and museums, there was some use of these venues by respondents, although only a minority were frequent users; 33% said they went more than once a month to gardens, 24% to libraries and 7% to museums (when not in lockdown).

**TABLE 1:** Meaning of the terms ‘culture’ or ‘the cultural sector’ to respondents

We asked respondents to write words or phrases that came to mind when hearing the terms ‘cultural sector’ or ‘culture’. They mentioned venues (museums, libraries, theatres, gardens and outdoor spaces, stately homes and castles, faith buildings, galleries, cinemas, markets), activities (art, opera, crafts, countryside walks and woodland trails, singing, dancing, amateur dramatics, sports, writing, photography, poetry, drawing and painting), events (exhibitions, lectures and talks, picnics, tourism and sightseeing, concerts), or entities (books, films, food, music, sculptures). Answers also referred to potential benefits that may occur from engaging with cultural institutions or activities: Entertainment/escapism Creativity/expression/freedom to be yourself/enriching life Mindful experience Learning/knowledge/educational/broadening the mind Connection/meeting with like‐minded people/sharing the experience of being human

Perceptions and personal use of cultural venues could influence whether they were considered as appropriate for older people. If link workers personally used museums before the pandemic (Χ^2^ [3, *N* = 109] = 10.74, *p* = 0.01) and during it (Χ^2^ [3, *N* = 148] = 16.18, *p* < 0.001) they were more likely to refer older people to such an organisation. It was also found that link workers were more likely to refer older people to gardens during the pandemic if they themselves used them during this time (Χ^2^ [3, *N* = 148] = 15.68, *p* < 0.001). However, these results are suggestive (due to the small numbers included in each comparison) and warrant further investigation.

### 3.2 Use of the cultural sector for older people within social prescribing

**FIGURE 1:**
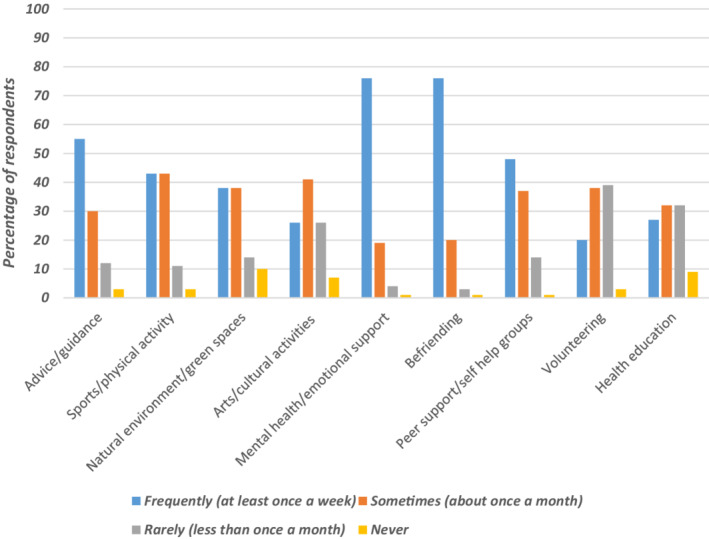
How often do you refer older people, as part of social prescribing, to the following?

Compared to other support, especially mental health and befriending services, arts and cultural offers were little used by link workers for older people (see Figure 1). Open comments on the questionnaire suggested this may be due to lack of such provision in a local area and costs. This relates to the dimensions of ‘affordable’ and ‘available’ in the European Patients Forum's accessibility classification. Similarly, the perception of cultural venues not being for all (i.e. elitist) can be related to ‘appropriate’ in this classification. Using local trusted community or faith groups was one means proposed by respondents to break down barriers that people may associate with the cultural sector not being for them.

**FIGURE 2:**
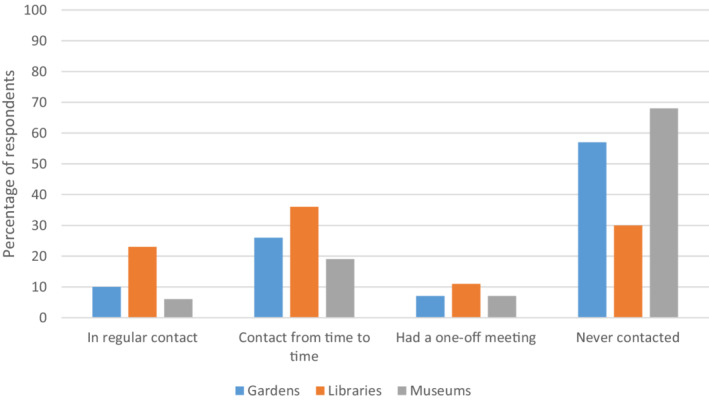
How often respondents approached venues about social prescribing offers/activities.

**FIGURE 3:**
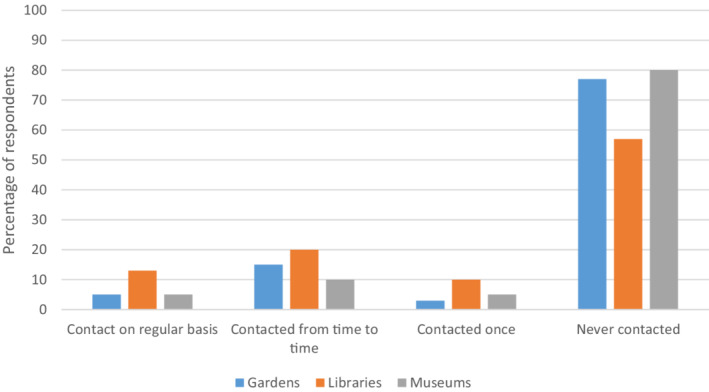
How often respondents are approached by venues about social prescribing offers/activities.

A lack of interaction between link workers and cultural sector staff could also be an issue. Questionnaire respondents called for better communication: *“both ‘sides’ need to connect, understand what each other do, support each other in their aims.”* Figure 2 highlights that respondents were more likely to be in contact with libraries about offers for social prescribing compared to gardens and museums. Three quarters had never approached or just had a one off meeting with museums, and two thirds had never approached or just had a one off meeting with gardens. Figure 3 shows that link workers who took part were rarely, if ever, contacted by any of these venues about available cultural offers. In terms of the European Patients Forum's classification, this lack of interaction could be problematic in link workers knowing if cultural offers are ‘adequate’ and ‘available’.

Link workers who approached gardens, libraries or museums about potential cultural offers were more likely to refer to such venues than those who had no contact or only one‐off contact (gardens: Χ^2^ [2, *N* = 148] = 18.24, *p* < 0.001; libraries: Χ^2^ [2, *N* = 148] = 20.87, *p* < 0.001; museums: Χ^2^ [2, *N* = 148] = 23.94, *p* < 0.001). Similarly, a significant association was found between frequency of link workers being approached by a garden, library or museum about such offers and referring older people to these venues, with those approached repeatedly more likely to refer (gardens: Χ^2^ [2, *N* = 148] = 15.29, *p* < 0.001; libraries: Χ^2^ [2, *N* = 148] 7.10, *p* = 0.03; museums: Χ^2^ [2, *N* = 148] = 20.01, *p* < 0.001).

### 3.3 COVID‐19 and use of gardens, libraries and museums for social prescribing with older people

Table 2 highlights how referring older people to public gardens remained relatively consistent during the pandemic. In contrast, there was a decline in referral to libraries and to museums; just over a quarter of respondents were referring older people to libraries and only one in 10 to museums. This is to be expected given that social distancing was in place and these venues were temporarily closed; this relates to ‘available’ in the European Patients Forum's classification. Respondents commented that a lot of community activities stopped running due to lockdown restrictions. Some were replaced with online services, requiring the use of a computer and the internet. It was noted that not all older people had digital access, they may not be interested in such an approach, or could struggle with using a computer, so were at risk of becoming digitally excluded. Issues related to ‘affordable’ and ‘appropriate’, as listed in the European Patients Forum's classification of accessibility, are pertinent here.

**TABLE 2:** Connection of older people by respondents to venues pre and during the pandemic

	How often they referred before the COVID‐19 pandemic		How often they referred during the COVID‐19 pandemic	
	Frequently or sometimes	Rarely or never	Frequently or sometimes	Rarely or never
Public gardens	43% of respondents	57% of respondents	38% of respondents	62% of respondents
Libraries	54% of respondents	46% of respondents	29% of respondents	71% of respondents
Museums	31% of respondents	69% of respondents	11% of respondents	89% of respondents

Longer time in post as a link worker was related to a higher number of referrals to gardens (Χ^2^ [6, *N* = 109] = 32.05, *p* < 0.001), libraries (Χ^2^ [6, *N* = 109] = 59.94, *p* < 0.001) and museums (Χ^2^ [6, *N* = 109] = 34.95, *p* < 0.001) during the pre‐COVID period. In contrast, no relationships were found between time as a link worker and referrals to these venues during the COVID pandemic. However, these results must be interpreted with caution. Respondents could indicate if they *were not* employed as a link worker prior to COVID; individuals who did this were excluded from the analyses meaning the sample size reduced to 109. Owing to the small sample size, multiple cells in the crosstabs table had counts of less than 5, so the reliability of the results is not assured.

### 3.4 Barriers and benefits to using gardens, libraries and museums for older people within a social prescription

**FIGURE 4:**
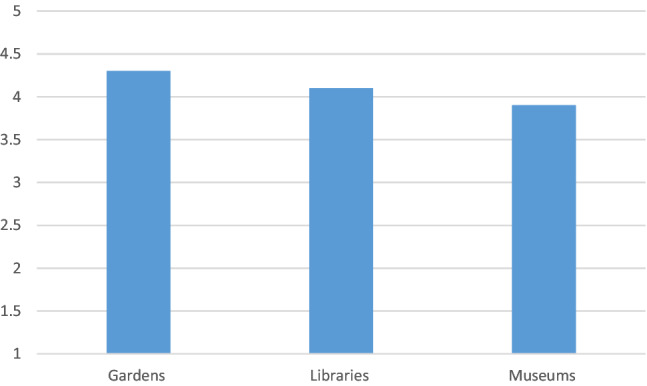
Average agreement from respondents with the statement that each of the following could support older people's well‐being—Gardens, libraries, museums—Rating from 1 (completely disagree) to 5 (completely agree).

There was a general sense from respondents' answers that these venues could be beneficial to older people's well‐being (see Figure 4).

Alongside perceived benefits, participants also reflected on the barriers that could arise to the use of these venues by older people as part of social prescribing. These are summarised in Table 3. Some barriers mentioned were physical (e.g. transport, accessible spaces) and relate to ‘accessible’ within the European Patients Forum's classification; others were more psychological (e.g. not being accustomed to these settings, lacking confidence to go alone), which can be related to ‘appropriate’ within this classification.

**TABLE 3:** Benefits and barriers for older people using gardens, libraries and museums as part of social prescribing

	Benefits	Barriers
Gardens	Being in the fresh air, with nature, encourages people to get out A calming environment that enables people to be present and reflect Connecting through group activities	Uneven pathways Costs Lack of transport Lack of seating
Libraries	Accessing books and having internet access for free (including through mobile libraries) Offering a quiet space Providing information about community events Connecting through group activities	Unfamiliarity (feeling out of place) Lack of interest
Museums	Learning opportunities that stimulate thinking (through talks, tours, activities, exhibitions) Venues that are beautiful and allow for reflection Connecting through group activities or volunteering	Costs Unfamiliarity (feeling out of place) Lack of transport Lack of accessible facilities

## 4 DISCUSSION

To the authors' best knowledge, this was the first attempt to explore, using a questionnaire, the views and experiences of link workers on the cultural sector within social prescribing, particularly for older people. The richness of responses to open‐ended questions created a large amount of data, suggesting an interest in the topic among link workers. Respondents recognised the potential of gardens, libraries and museums as part of social prescribing but did not necessarily refer people to these venues, especially during the pandemic. This may have been due to the closure of these venues during lockdowns. Alternatively, it may have been because they were unfamiliar with such settings. Therefore, increasing connections between cultural sector staff and link workers seems important, and may require investment to allow mutual understanding to be fostered (e.g. having time for joint meetings, setting up taster sessions for link workers of cultural offers, developing newsletters, running social media groups). These issues relate to dimensions of accessibility listed in the European Patients Forum's classification of this concept.

Literature about arts‐on‐prescription and museums‐on‐prescription, as particular forms of social prescribing, emphasises that such initiatives can support people with poor mental health or experiencing psychosocial distress or feeling social isolated (Bungay & Clift, 2010). It has been noted that cultural and heritage venues are ideally placed within communities to support such delivery (Thomson et al., 2015). However, in line with issues raised about accessibility by the European Patients Forum's classification of this concept, questionnaire data suggested a need for some link workers to see the potential of the cultural sector as a social prescribing option. Cultural venues may have to be proactive in presenting information about what they can provide and how they welcome individuals from all backgrounds. They may have to help people overcome issues around psychological accessibility (e.g. not feeling they are someone who would fit into a cultural venue, lacking confidence to attend cultural activities). Other research in this area suggests that a ‘buddy’ system could be one way of addressing such challenges (Tierney et al., 2022). Likewise, cultural venues must consider physical accessibility (e.g. transport, accessible toilets, adequate seating, clear signage) to make venues feel like welcoming spaces to all.

Running outreach sessions, away from imposing buildings, may be another means of making cultural activities appealing and accessible to a wider range of older people (Duncan, 2018; Greaves & Farbus, 2006; Thompson et al., 2020). These buildings were closed during the pandemic, but online or mobile provision was offered as an alternative, which enabled people to escape from their concerns through absorbing activities and to feel connected to the outside world (Art Fund, 2020; Kaplan, 2020; Price, 2021). However, digital exclusion may have prevented some older people from accessing these resources (Age UK, 2021). This was a concern raised by respondents to the questionnaire and is another issue that relates to accessibility as defined by the European Patients Forum's classification. Recent efforts have been established to provide people with technology and/or training in online communication (Age UK, 2022; Connecting Scotland, 2021).

### 4.1 Future research

Results from exploratory analyses could be examined further in future research. For example, a relationship between time in post and frequency of referral to the cultural sector could reflect a link worker's increased knowledge of available local resources over time. This may include having the opportunity to meet with providers or to visit venues themselves; exploring the benefits through taster sessions specifically for link workers is warranted. Responses suggested that familiarity with a venue might be linked to increased referral of older people to it. An explanation for this could be that link workers feel safe in making such a referral because they have had first‐hand experience of what to expect so can suggest it with confidence. Future research could also explore how link workers make decisions on who to propose a cultural offer to and why; this may help to reduce any perpetuation of stereotypes in terms of who is connected to such provision (and who is not given this opportunity).

### 4.2 Limitations

It should be noted that the analyses of association were post‐hoc comparisons; they were not part of the planning for the research or the sampling. It should also be borne in mind that the study was conducted during the COVID‐19 pandemic, towards the end of a third lockdown period in the UK (April–May 2021). It was distributed after a year of readjustment, as the cultural sector responded to social restrictions and changes in the way it delivered cultural offers.

Responses came from 148 link workers. They were recruited via a range of routes, but represent a proportion of total link workers employed in the UK (mainly from England). It should be acknowledged that this was a self‐selected sample who opted to complete the online questionnaire, who may have had some interest in the cultural sector.

## 5 CONCLUSION

Link workers responding to the questionnaire, overall, supported the idea of the cultural sector contributing to older people's well‐being. However, this was not necessarily reflected in their referral behaviours to gardens, libraries or museums. In part, this may be due to a lack of contact or familiarity with such venues, and preconceptions of how cultural offers would be regarded by older people. Better connection between link workers and the cultural sector may be required for the latter to play a substantive role in social prescribing. Furthermore, offers provided by this sector for older people need to address physical and psychological barriers identified in the questionnaire data. The questionnaire highlighted the importance of addressing issues related to accessibility. This will ensure that a range of individuals can draw on the benefits that can transpire from engaging with cultural offers, which will be useful for link workers as they assist people in need during the recovery period from the pandemic and beyond.

## AUTHOR CONTRIBUTIONS

ST, KRM, GW, EW, LS, HJC, KH, CP, KE were involved in the design of the project. SL, JG, OA, ST and CP were involved in data collection and analysis. All authors were involved in interpretation of data. All authors were involved in developing this paper.

## CONFLICT OF INTEREST

The authors have no conflicts of interest to report.

## Data Availability

Research data are not shared.
